# Astrocytic Calcium and cAMP in Neurodegenerative Diseases

**DOI:** 10.3389/fncel.2022.889939

**Published:** 2022-05-19

**Authors:** Marta Sobolczyk, Tomasz Boczek

**Affiliations:** Department of Molecular Neurochemistry, Faculty of Health Sciences, Medical University, Lodz, Poland

**Keywords:** astrocyte, calcium, cyclic AMP (cAMP), neurodegeneration, Parkinson's disease, PKA, adenylyl cyclases, Alzheimer's disease

## Abstract

It is commonly accepted that the role of astrocytes exceeds far beyond neuronal scaffold and energy supply. Their unique morphological and functional features have recently brough much attention as it became evident that they play a fundamental role in neurotransmission and interact with synapses. Synaptic transmission is a highly orchestrated process, which triggers local and transient elevations in intracellular Ca^2+^, a phenomenon with specific temporal and spatial properties. Presynaptic activation of Ca^2+^-dependent adenylyl cyclases represents an important mechanism of synaptic transmission modulation. This involves activation of the cAMP-PKA pathway to regulate neurotransmitter synthesis, release and storage, and to increase neuroprotection. This aspect is of paramount importance for the preservation of neuronal survival and functionality in several pathological states occurring with progressive neuronal loss. Hence, the aim of this review is to discuss mutual relationships between cAMP and Ca^2+^ signaling and emphasize those alterations at the Ca^2+^/cAMP crosstalk that have been identified in neurodegenerative disorders, such as Alzheimer's and Parkinson's disease.

## Introduction

The discoveries made three decades ago that cytosolic Ca^2+^ rise occurs in astrocytes in response to environmental cues have provided new insights into the essential role of these star-shaped glial cells in the CNS. Nowadays, it is commonly known that astrocytes are highly specialized types of glial cells responsible for synapse formation and regulation of ongoing neuronal transmission. They also interact with other glial cells or blood vessels depending on the brain region (Verkhratsky and Nedergaard, 2018). For instance, the astrocytic support is maintained by the release of neurotrophic factors and diverse transmitters called gliotransmitters, including adenosine triphosphate (ATP)/adenosine, D-serine, glutamate, tumor necrosis factor α (TNFα), and γ-aminobutyric acid (GABA) at various timescales to sustain complex information processing and metabolic homeostasis in the brain (Steeland et al., 2018; Durkee and Araque, 2019; Pöyhönen et al., 2019). It is estimated that the ratio of glial cells to neurons is roughly 1:1 and astrocytes constitute ~19–40% of all glial cells in the CNS (Verkhratsky and Nedergaard, 2018), nevertheless the total number of neurons and glia has long been controversial (von Bartheld et al., 2016).

The morphological and functional heterogeneity of astrocytes determines various protein expression profiles what may explain the sensitivity of certain areas of the brain to the progress of a specific disease entity (Xin and Bonci, 2018; Matias et al., 2019). Moreover, opposed to the neuro-centric view of brain function, astroglia dysfunction is increasingly considered a fundamental cause in the pathogenesis of neurological diseases (Liu et al., 2017; Robertson, 2018).

In contrast to neurons, astrocytes do not fire an action potential. However, in response to the local changes in intracellular Ca^2+^, often referred to as “Ca^2+^ excitability,” they can strongly influence physiological and pathophysiological events in the nervous system. Indeed, astrocytes express the diversity of G protein-coupled receptors (GPCRs) that allow detection and reaction to neuronal signals (Durkee and Araque, 2019). Among the second messengers activated by GPCRs, Ca^2+^, and cyclic adenosine monophosphate (cAMP) are capable of eliciting diverse pleiotropic responses, thus regulating basic cellular functions, such as growth and differentiation, gene transcription and protein expression as well as astrocyte-mediated synaptic plasticity, gliotransmission, energy supply and maintenance of the extracellular environment (Bazargani and Attwell, 2016; Horvat and Vardjan, 2019; Zhou et al., 2021). It is supposed that dysregulation of Ca^2+^ and cAMP exacerbates structural and functional abnormalities in this cell type, hence restoration of imbalanced Ca^2+^ and/or cAMP signaling may constitute an effective astrocyte-based therapeutic approach Growing body of evidence links deficits in astrocytic Ca^2+^ and cAMP-controlled mechanisms to various brain pathologies (Ujita et al., 2017; Reuschlein et al., 2019; Kofuji and Araque, 2021; Zhou et al., 2021). Recent technological progress in two-photon imaging and development of genetically encoded Ca^2+^ indicators (GECIs) as well as genetically encoded sensors for cAMP and protein kinase A (PKA), allowed for high-resolution detection of astrocytic Ca^2+^/cAMP fluxes in different physiological and pathological conditions (Reeves et al., 2011; Gee et al., 2015; Semyanov et al., 2020; Massengill et al., 2021).

Despite this progress, the cause-effect relationship between temporal and spatial disturbances in intracellular Ca^2+^/cAMP signaling machinery and the development of neuropathological disorders are still being sought. Therefore, this review summarizes the latest findings on the crosstalk between cAMP and Ca^2+^ signaling pathways and their contribution to the neurodegenerative process.

### Ca^2+^/cAMP in Astrocyte Homeostasis: A Brief Review

**Figure 1:**
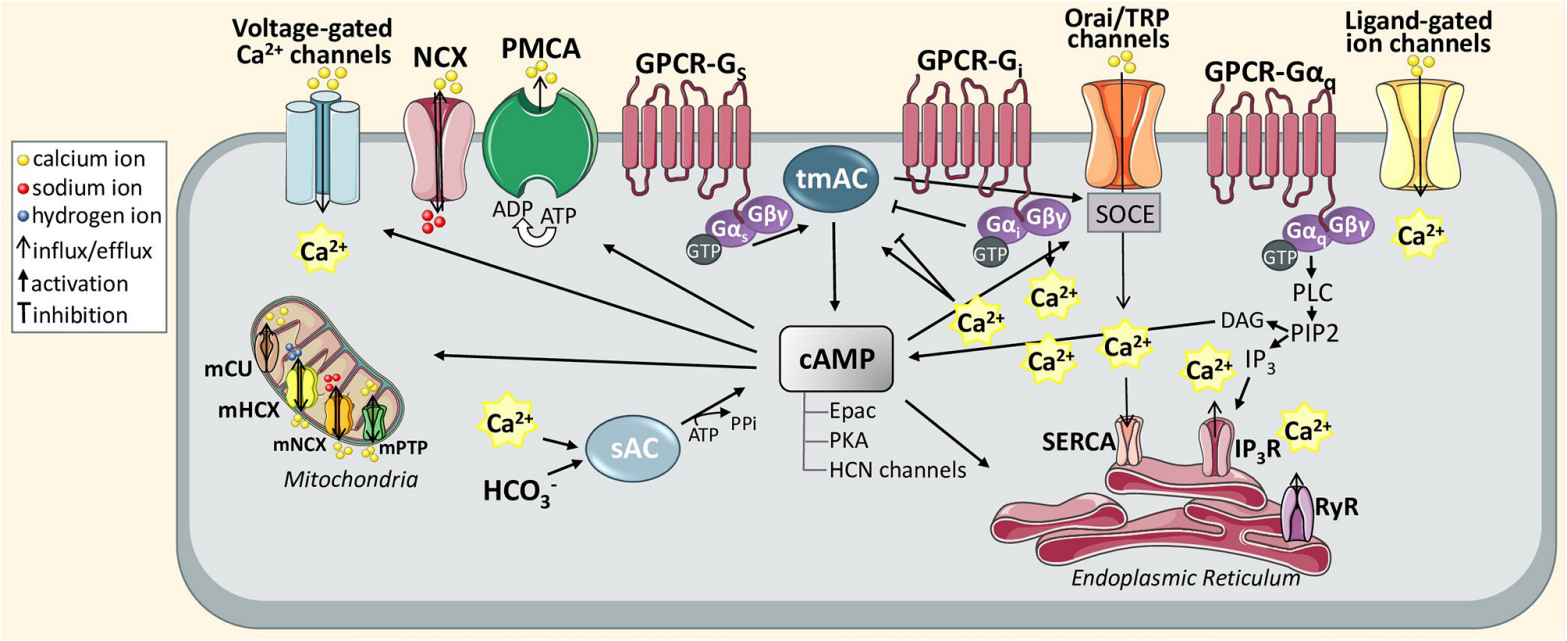
Schematic diagram of astrocytic Ca^2+^ and cAMP signaling pathways discussed in this review. The action of active GTP-bound Gα_i_ and GTP-bound Gα_o_ stimulates or inhibits transmembrane adenylyl cyclase (tmAC) that produces cAMP. The tmAC may also be controlled indirectly *via* store-operated Ca^2+^ entry (SOCE). To maintain Ca^2+^ homeostasis in the ER, SOCE is orchestrated through the interaction between store-operated plasma membrane calcium channels, called Orai or transient receptor potential (TRP) channel, and can stimulate SERCA pump activity once Ca^2+^ levels fall below the threshold levels. In particular, this mechanism is generated when Ca^2+^ stores in the ER are depleted upon activation of inositol 1,4,5-trisphosphate (IP3) receptor via Gα_q_-phospholipase C (PLC)-IP3 signal transduction pathway. In turn, the DAG produced from PIP2 hydrolysis can also catalyze cAMP synthesis. Another activator of cAMP is Ca^2+^ sensitive soluble adenylyl cyclase (sAC). cAMP itself and cAMP-dependent proteins (Epac PKA, and HCN channels) can differently transmit Ca^2+^ signals supporting various astrocytic functions including gliotransmission, glycogen metabolism or synaptic homeostasis.

Overall, neuronal information is transferred to astrocytes primarily through spillover of neurotransmitters or other types of neuroligands, which diffuse into the extracellular space and next bind to various astroglial targets, such as membrane ionic channels, transporters or receptors triggering their conformational change (Figure 1). As a result, activated GPCR catalyzes dissociation of a heterotrimeric G protein complex (composed of G_α_, G_β_/G_γ_ subunits) into G_α_ subunit and βγ dimer by the exchange of GDP for GTP. Based on the sequence homology and functionality of α-subunits, G proteins are divided into four main families: G_α*s*_, G_α*i*_/G_α*o*_, G_α*q*_/G_α11_, and G_α12_/G_α13_ that regulate distinct downstream signaling events (Jastrzebska, 2013). The activation of the G_q_ subunit stimulates phospholipase C (PLC) that leads to hydrolysis of phosphoinositol diphosphate (PIP2) into diacylglycerol (DAG), known as a membrane-bound regulator of cAMP concentration, and inositol 1,4,5-triphosphate (IP3), known as a soluble messenger triggering the release of Ca^2+^ ions from the endoplasmic reticulum (ER) (Hua et al., 2004) or secretory vesicles (Hur et al., 2010). In astrocytes, G_q_-coupled receptors, mainly α1-adrenoreceptor (α_1_-AR; Ding et al., 2013), D1 dopamine receptor (D1R; Corkrum et al., 2020), histamine receptor (H_1_; Kárpáti et al., 2018), metabotropic glutamate receptor (mGluR; Sun et al., 2013), serotonin 5-HT_2_ receptor (Peng and Huang, 2012), and P2Y purinoreceptor (Ding et al., 2009), and also G_i_-coupled receptors, such as GABA_B_ receptor (Durkee et al., 2019), generate a wide range of inositol 1,4,5-trisphosphate receptor (IP3R)-dependent Ca^2+^ oscillations to differently regulate gliotransmission and neuronal modulation (Table 1). For example, synaptically released acetylcholine (ACh) or noradrenaline (NE) can induce an astrocytic Ca^2+^ increase thereby enhancing synaptic plasticity the in the cortex (Takata et al., 2011; Chen et al., 2012) and hippocampus (Navarrete et al., 2012; Papouin et al., 2017). The IP3R-dependent Ca^2+^ release may also occur spontaneously (King et al., 2020). The canonical function of G_i_-GPCR is to suppress adenylate cyclase-dependent signaling cascade and thus, inhibit cAMP activity, which has been also observed in astrocytes (Gould et al., 2014). The G_βγ_ released from different G_i_ subunits targets ion channels such as inwardly rectifying potassium channels and voltage-gated Ca^2+^ channels (Jeremic et al., 2021). Interestingly, the GPCR-G_i/o_ protein signaling in neurons is commonly known to inhibit intracellular Ca^2+^ events and electrical excitability, whereas a recent study has demonstrated that astrocyte G_i/o_ GPCR activation may stimulate Ca^2+^ elevation involved in the release of inhibitory neurotransmitters into the synapse (Huang and Thathiah, 2015; Durkee et al., 2019). Therefore, such functional diversity of GPCRs enables integrated astrocyte-neuron communication.

**Table 1:** Astrocytic Ca^2+^/cAMP modulation by GPCR receptors and their physiological role in diverse brain areas.

**GPCR(s)**	**G protein(s)**	**Effect on 2nd messenger**	**Brain region**	**Gliotransmission**	**Psychological role**	**References**
A_1_R	G_i/o_	↓cAMP	Cortex	Glutamate	Modulation of synaptic transmission	Cristóvão-Ferreira et al., 2013
A_2A_R	G_s_	↑cAMP	Cortex	-	Enhancement of GABA uptake	Cristóvão-Ferreira et al., 2013
α_1_AR	G_q_	↑Ca^2+^	Cortex	ATP/D-serine	Control of synaptic plasticity	Pankratov and Lalo, 2015
α_2_AR	G_i/o_	↓cAMP	Cortex	-	-	Kitano et al., 2021
βAR	G_s_	↑cAMP	Cortex	-	-	Horvat et al., 2016; Kitano et al., 2021
βAR	G_s_	↑cAMP	-	-	Glucose uptake	Catus et al., 2011
D1R	G_q_	↑Ca^2+^	Nucleus Accumbens	ATP/adenosine	Depress excitatory synaptic transmission	Corkrum et al., 2020
D1/5R	G_S_	↑cAMP	Cortex	-	Mediation of intracellular NADH increase	Requardt et al., 2010
GABA_B_R	G_i/o_	↑Ca^2+^	Cortex	Glutamate	Increase neuronal excitability	Mariotti et al., 2016
GABA_B_R	G_i/o_	↑Ca^2+^	Striatum	Thrombospondin-1	Increase synaptic excitability and transmission	Nagai et al., 2019
H_1_R	G_q_	↑Ca^2+^	Cortex	Glutamate	-	Kárpáti et al., 2018
H_2_R	G_s_	↑cAMP	-	-	-	Kárpáti et al., 2018
mGluR2	G_i/o_	↑Ca^2+^	Thalamus	Glutamate	Synaptic inhibition	Copeland et al., 2017
mGluR3	G_i/o_	↓cAMP	Cortex	-	Protection against hypoxic/ischemic damage	Ciccarelli et al., 2007; Sun et al., 2013
mGluR5	G_q_	↑Ca^2+^	Hippocampus	ATP	Enhance basal synaptic transmission	Panatier et al., 2011
P2Y1R	G_q_	↑Ca^2+^	Hippocampus	Glutamate	Increase synaptic potentiation	Santello et al., 2011; Álvarez-Ferradas et al., 2015
P2Y1R	G_q_	↑Ca^2+^	Cortex	ATP	Modulation of synaptic plasticity	Lalo et al., 2019
PAC1R	G_s_	↑cAMP	Cortex	Endozepine	Activate neuronal metabotropic receptor	Masmoudi-Kouki et al., 2007
5-HT_2_	G_q_	↑Ca^2+^	-	Glutamate	Increase neuronal excitation	Chen et al., 2016
5-HT_4_R	G_s_	↑cAMP	-	-	Regulation of immune responsiveness	Zeinstra et al., 2006
5-HT_5A_R	G_i/o_	↓cAMP	-	-	-	Carson et al., 1996
MOP	G_i/o_	↓cAMP	Hippocampus	-	Decrease neuronal excitability	Machelska and Celik, 2020

*↑Means increased*.

*↓Means decreased*.

*-Means not detected or not defined*.

Several interesting results regarding global Ca^2+^ elevations in astrocytes have been derived from IP3R type 2 knockout mice (Guerra-Gomes et al., 2021). It has been demonstrated that loss of this receptor may contribute to various types of cognitive dysfunctions (Perez-Alvarez et al., 2014; Padmashri et al., 2015) as well as depressive-like behaviors (Cao et al., 2013), but it seems unlikely that its role in shaping astrocytic Ca^2+^ is predominant (Petravicz et al., 2008, 2014). Although astrocytic IP3R2 expression is required for certain mechanisms of LTD formation, the genetic defects in IP3R2 are not sufficient to fully prevent LTP generation what probably reflects astrocyte diversity in different brain regions (Oberheim et al., 2012). Recently, it has been suggested that IP3R1 and IP3R3 subtypes co-exist in astroglia and retain their functionality by generating local Ca^2+^ events (Sherwood et al., 2017, 2021). The second key type of Ca^2+^-permeable receptor channel in the ER is the ryanodine receptor (RyR) but the mechanism of its activation and inhibition by Ca^2+^ remains largely unexplored and controversial (Rodríguez-Prados et al., 2020; Skowrońska et al., 2020). The latest report has suggested that RyR-mediated Ca^2+^-induced Ca^2+^ release (CICR) in astrocytes may be negatively modulated by neuron-derived factors which may alter the Ca^2+^ response triggered by ionotropic receptors (Skowrońska et al., 2020). Other types of stores controlling intracellular Ca^2+^ concentration are mitochondria that buffer cytosolic Ca^2+^ *via* the mitochondrial NCX, mitochondrial H^+^/Ca^2+^ exchanger (mHCX), mitochondrial Ca^2+^ uniporter (mCU), and mitochondrial permeability transition pore (mPTP; Agarwal et al., 2017). Interestingly, the expression of some mitochondrial transporters may be controlled by cyclic AMP response element-binding protein (CREB; Shanmughapriya et al., 2015). Primarily, a rise in mitochondrial Ca^2+^ concentration drives energy production needed to modulate glutamate release and prevents excitotoxic neuronal death. On the other hand, Ca^2+^ overload may lead to astrocytic apoptosis (Stephen et al., 2014). It is worth mentioning that Ca^2+^ signaling dynamics diffuse between mitochondria and ER (Okubo et al., 2019), and ER stress triggers mitochondrial dysfunction (Britti et al., 2018).

The resting cytosolic Ca^2+^ concentration independent from IP3-activity is also controlled by transmembrane Ca^2+^ influx through the transient receptor potential A1 (TRPA1) channel. Activation of this receptor can induce constitutive D-serine release from astrocytes for NMDA receptor-dependent LTP maintenance (Shigetomi et al., 2013). Blocking of TRPA1 decreases spontaneous Ca^2+^ events leading to lower extracellular GABA uptake by the astrocyte-specific transporter (GAT3; Shigetomi et al., 2011). In view of that, astrocytic TRPA channels are considered as integral players coordinating Ca^2+^ dynamics involved in the inhibitory efficacy of the hippocampal synapses. Likewise, intracellular Ca^2+^ is also tightly regulated by ionotropic receptors, including α-amino-3-hydroxy-5-methyl-4-isoxazolepropionate (AMPA) and N-methyl-D-aspartate (NMDA) receptors, both gated by glutamate, and ATP-gated purinergic P2X receptors (Palygin et al., 2010, 2011; Ceprian and Fulton, 2019). The plasma membrane Na^+^/Ca^2+^ exchanger (NCX) and plasma membrane Ca^2+^ ATPase (PMCA) are the main regulators of Ca^2+^ extrusion to the extracellular space while the control of Ca^2+^ concentration in the ER is ensured by the sarco/endoplasmic reticulum calcium ATPase (SERCA) (Brini and Carafoli, 2011). Compared to NCX, PMCA has a lower capacity for ionic transport but its basal affinity for Ca^2+^ is higher. This property allows PMCA to respond to even subtle changes in Ca^2+^ concentration within a range of its resting cytosolic level. By contrast, NCX can eliminate significant rises in intracellular Ca^2+^, although, in the case of increased intracellular Na^+^ concentration, due to e.g., glutamate or GABA uptake, the NCX may operate in reverse mode increasing Ca^2+^ influx (Brini and Carafoli, 2011; Rose et al., 2020).

Unlike Ca^2+^ activity, which can be regulated by multiple distinct pathways, the predominant mechanism of cAMP modulation is mediated by GPCRs coupled to either G_s_ or G_i_ leading to activation or inhibition of transmembrane adenylate cyclases (tmACs), respectively. In astrocytes, cAMP is regulated mainly by the activation of adenosine receptors (A_1_ and A_2A_), adrenergic receptors (α_2_ and β_1−3_), dopamine receptor (D1/5), glutamatergic receptor (mGlu3), histamine receptor (H_2_), PACAP/VIP receptors, serotonin receptors (5-HT_4_ and 5-HT_5A_) and also opioid receptors, all summarized in Table 1. ACs are believed to be pivotal points of integration between Ca^2+^ and cAMP signaling. It is assumed that all of the nine tmAC isoforms, which have also been identified in mice astrocytes (Lee et al., 2018), can be modulated (activated or inhibited) by Ca^2+^, either directly or indirectly *via* Ca^2+^ binding proteins such as calmodulin (CaM), CaM kinase (CaMK), calcineurin (CaN), protein kinase C (PKC), or G_q_-coupled receptor activation. For instance, AC8 activated in a Ca^2+^/CaM-dependent manner binds the Orai1 channel, which is a major functional component responsible for store-operated calcium entry (SOCE; Willoughby et al., 2012a,b). Accordingly, the AC8 seems to be highly sensitive to modest local Ca^2+^ changes. In primary astrocytic cultures, AC inhibitor 2',5'-dideoxyadenosine prevented cAMP synthesis and significantly decreased SOCE-triggered glycogenolysis. Based on this evidence, the authors proposed a new model for astrocytic coupling of Ca^2+^ homeostasis, AC8-dependent cAMP production and glycogen metabolism which could also impact on learning and memory processes (Müller et al., 2014). However, the physiological interplay between the molecular players of cAMP signaling and depletion of ER Ca^2+^ stores still remains to be determined in this subtype of glial cells.

Recent *in vitro* and *in vivo* studies provided considerable insight into the differences between Ca^2+^ and cAMP dynamics in astrocytes in terms of precise spatiotemporal regulation of complex cellular processes (Horvat et al., 2016; Oe et al., 2020). While stimulation of α_1_AR generated rapid and transient Ca^2+^ increase enhancing synaptic plasticity, stimulation of βAR triggered slower and long-lasting cAMP elevations and promoted consolidation of cortical memory. It is supposed that threshold levels for activation of respective second messenger are different due to diverse affinities of NE for AR subtypes. According to this, moderate NE release may be sufficient to activate the α_1_AR coupled to the G_q_ while activation of βAR coupled to the G_s_ needs relatively high extracellular NE to elevate cAMP within the cell (Ramos and Arnsten, 2007; Oe et al., 2020). Another type of AC, called soluble AC (sAC), is directly activated by ${\text{HCO}}_{3}^{-}$ entry *via* the electrogenic NaHCO_3_ cotransporter in response to extracellular K^+^ rises or aglycemia (Choi et al., 2012; Schmid et al., 2014). sAC is also highly expressed in astrocytes to stimulate the production of intracellular cAMP. Elevated cAMP can provide energy supply and metabolic support for the proper functioning of neurons contributing to glycolysis and delivery of lactate from astrocytes (Choi et al., 2012; MacVicar and Choi, 2017). It is worth noting that sAC is found in distinct subcellular microdomains with local cAMP signals (Tresguerres et al., 2011) and even slight changes in the intracellular Ca^2+^ dynamics may impact the activation of this AC isoform (Gancedo, 2013; Schmid et al., 2014). The fatty acids (Lee et al., 2018), lactate, aspirin (Modi et al., 2013), or some antidepressants (e.g., ketamine or fluoxetin) have been reported to cause the astrocytic cAMP-elevation but their mechanism of action toward second messengers still remains unclear (Kinoshita et al., 2018; Lasič et al., 2019; Stenovec et al., 2020; Stenovec, 2021).

It is commonly known that Ca^2+^ has multiple downstream targets (Scemes and Giaume, 2006; Bagur and Hajnóczky, 2017). cAMP can transmit Ca^2+^ signals through isoforms of exchange protein activated by cAMP (Epac), Epac1 and Epac2, or PKA or cAMP-gated ion channels called hyperpolarization-activated cyclic nucleotide-gated (HCN) channels (Halls and Cooper, 2011). cAMP itself or cAMP-dependent proteins may evoke both Ca^2+^ influx *via* cation channels (Catterall, 2011) and Ca^2+^ extrusion by ATP-dependent pumps (Vandecaetsbeek et al., 2011). Phosphorylation of IP3R subtypes seems to be stimulated by PKA or EPAC as well as directly activated by cAMP. This activation requires much higher concentrations of second messengers and is quickly attenuated after removing the AC stimulus (Taylor, 2017). Analysis of acute hippocampal slices has shown that astrocytic cAMP/PKA signaling may modulate oscillatory activity of intracellular Ca^2+^ (Ujita et al., 2017). In cortical astrocytes, cAMP/PKA-dependent Ca^2+^ changes may be mediated by voltage-gated Ca^2+^ channels (VGCCs) to maintain the exocytotic secretion of gliotransmitters (Burgos et al., 2007). The cytosolic Ca^2+^ elevations are necessary for gliotransmission of ATP, serine, and also glutamate to support neuronal plasticity (Harada et al., 2015). In pathological states, the abnormal release of gliosignalling molecules may trigger excitotoxicity and synaptic damage leading to neuroinflammatory and neurodegenerative progress (Agulhon et al., 2012; Kawamata et al., 2014).

Both cAMP and cGMP degradation may be driven by a large group of phosphodiesterases (PDEs) classified into 11 families, some of which are regulated by Ca^2+^ or Ca^2+^/CaM (Bender and Beavo, 2006; Tenner et al., 2020; Turunen and Koskelainen, 2021). Given that most of tmAC isoforms, PKA and cyclic nucleotide phosphodiesterases (PDEs), may be located at the scaffold protein complexes called A-kinase anchoring proteins (AKAPs), it is plausible that the spatial and temporal organization of AKAPs orchestrates synthesis and degradation of the second messenger at the specific subcellular sites. Although RNA-sequencing of astrocytes purified from mouse brain suggests the presence of genes encoding various AKAP subtypes (e.g., AKAP15/18, AKAP79, gravin, Yotiao; Reuschlein et al., 2019), their functional role in astrocytic cAMP signaling remains to be uncovered. These multi-functional scaffold proteins have been widely characterized in neurons, both in physiological and pathological states (Wild and Dell'Acqua, 2018). The AKAPs associated with cAMP are regulated by local Ca^2+^ oscillations (Scott and Santana, 2010; Boczek et al., 2021). Global analysis of astroglial transcripts released in 2015 demonstrated that cAMP-dependent signaling regulated 6,221 of 16,594 annotated genes, including 42.1% of them were significantly upregulated whereas the remaining were downregulated by cAMP analogs. As suggested by Gene Ontology enrichment analysis, the upregulated genes were mainly involved in cellular metabolism (e.g., uptake/degradation of catecholamines, glutamate, and glycine) and transport (e.g., Ca^2+^ or K^+^ -ion, glucose, or water transport) as well as antioxidant activities, especially glutathione-related defenses. Among genes downregulated by cAMP stimulation, the overwhelming number of encoded modulators of the physiological process as cell cycle, proliferation, or death (e.g., some cyclins and cyclin-dependent kinases, mitogenic agents, BCL2 family proteins), as well as cytoskeletal proteins and mediators of the immune system. Therefore, cAMP is involved in the regulation of a plethora of cellular functions from the antioxidant systems through the control of the normal state of differentiated astrocytic cells (Paco et al., 2016).

It is apparently becoming clear that the existence of an interplay between cAMP and Ca^2+^ messenger systems in astrocytes is non-linear and of great complexity. Undoubtedly, spatial and temporal resolution should be taken under consideration to understand their synergistic and antagonistic relationship.

## Ca^2+^ and cAMP Signaling Pathways in the Neurodegenerative Diseases

The pathological protein accumulation, such as amyloid beta (Aβ) plaques and neurofibrillary tangles composed of hyperphosphorylated protein tau are commonly detected hallmarks of Alzheimer's disease (AD). Neurodegeneration manifested by neuronal integrity loss and white matter lesions or gliosis, is widely recognized in postmortem brain of AD patients (Raskin et al., 2015). During progression of AD, the pathological extra-neuronal Aβ deposits interrupt astrocyte's functions leading to disruption of gliotransmission, neurotransmitter uptake, and Ca^2+^ handling. The Aβ-induced astrocytic metabolic failure, including the production of reactive oxygen/nitrogen species, Ca^2+^-dependent glutathione depletion, nuclear factor kappa-light-chain-enhancer of activated B cells (NF-κB) activation and mitochondrial Ca^2+^ dyshomeostasis, is ultimately linked to synaptic dysfunction and neuronal death (Abramov et al., 2004; Abeti et al., 2011). Indeed, Aβ exposure impaired Ca^2+^ homeostasis *via* activation of Ca^2+^ sensitive channels or Ca^2+^ permeable ionotropic receptors leading to abnormal Ca^2+^ permeability, and subsequent cytosolic Ca^2+^ overload (Demuro et al., 2010). The application of Aβ oligomers was found to promote Ca^2+^ rise associated with ER stress response initiating reactive astrogliosis, which has been characterized both *in vitro* and *in vivo* (Alberdi et al., 2013). The development of astrocyte reactivity promotes molecular and morphological remodeling of astrocytes that may have neuroprotective or neurotoxic effects depending on the occurrence of pathological condition in the specific brain region. This phenomenon is observed mainly at the early stages of disease. Remodeled astrocytes usually exhibit changes in gene expression profile, increased level of cytoskeletal structural proteins (GFAP and vimentin), and cellular hypertrophy (Pekny et al., 2016). In animal models of AD, astroglial phenotype transformation emerged around Aβ deposits in the hippocampus, but the reactivity has not been reported in the entorhinal and prefrontal cortex (Verkhratsky et al., 2016). The pro-inflammatory bradykinin which is often elevated in the plasma of AD patients with greater cognitive disruption (Singh et al., 2020), may induce Ca^2+^ hyperactivity *via* nicotinic acetylcholine receptors (AChRs) and PI3K-Akt signaling pathway in cortical astrocytes cultures (Makitani et al., 2017). It is also worth mentioning that bradykinin is associated with increased NO production and induction of vascular permeability leading to disruption of the BBB barrier integrity (Erickson and Banks, 2013; Makitani et al., 2017).

Loss of dopaminergic neurons, the presence of α-synuclein and inclusion bodies in brain tissue are the hallmarks of Parkinson's disease (PD), the second most recognized neurodegenerative disorder. The α-synuclein proteins secreted from neurons are easily taken up by astrocytes through endocytosis causing inflammatory response such as release of cytokines (IL1, IL6, and TNFα), oxidative stress and mitochondrial impairment (Gu et al., 2010; Lee et al., 2010). Particularly, dopaminergic neurons in the substantia nigra pars compacta (SNpc) affect movement control as well as reward response and their progressive degeneration caused by chronic inflammation and reactive astrogliosis, is one of the main factors determining characteristic motor disturbance (e.g., bradykinesia, rigidity, tremor at rest) in PD patients (Michel et al., 2013). Generally, dopamine receptors are classified into two subfamilies, D1-like receptor family coupled to G_s/olf_ (consists of D1 and D5), and D2-like receptor family coupled to G_i/o_ (consists of D2, D3, and D4 receptors) to stimulate or inhibit AC/cAMP/PKA transduction pathway, respectively. All five subtypes of dopamine receptors are also expressed in astrocytes. In cultured cortical astrocytes, the application of SKF83959 led to discovery of non-cyclase-coupled D1-like receptor called phosphatidyl-inositol-linked D1 receptor which induces Ca^2+^ mobilization *via* the G_q_/PLC/IP3 signaling (Liu et al., 2009).

To date, there is no conclusive evidence on the crosstalk between cAMP and Ca^2+^ in PD pathology, but several reports suggest that mutual modulation of the second messengers in dysfunctional astrocytes likely contributes to neurodegeneration observed in this disorder. So, the following sections highlight this evidence pointing out potential mechanisms that could trigger abnormal Ca^2+^/cAMP signaling and thus promote loss of supporting astrocyte function.

### Effect on Glutamate Excitotoxicity and Neurotrophic Support

Astrocytic excitatory amino acid transporters (EAATs) defects have been repeatedly demonstrated in numerous neurodegenerative diseases, especially AD, PD but also Huntington's disease (Su et al., 2003; Sharma et al., 2019; Hindeya Gebreyesus and Gebrehiwot Gebremichael, 2020). Increased level of Aβ suppresses the expression of two important astroglial transporters: GLAST (glutamate-aspartate transporter, also named EAAT1) and GLT1 (glutamate transporter 1, also named EAAT2) *via* G_s_-coupled A_2A_ receptors decreasing glutamate uptake by astroglia (Matos et al., 2012; Zumkehr et al., 2015). Secondarily, adenosine A_2A_ receptor/PKA signaling pathway regulates intracellular Ca^2+^ mobilizations and triggers glutamate release. Remarkably, this pathway can stimulate the rise in cytosolic Ca^2+^ concentration *via* Ca^2+^ efflux from intracellular Ca^2+^ stores independent of IP3 and ryanodine receptor activity (Kanno and Nishizaki, 2012). In AD patients, the overexpression of adenosine A2A receptor gene (ADORA2A) is aggravated (Horgusluoglu-Moloch et al., 2017) which can lead to decreased ability of astrocytes to clear the extracellular glutamate (Matos et al., 2012). In addition to its central function as an energy source, glycogen is also essential to provide energy for glutamate and glutamine synthesis in response to elevated K^+^ concentrations or neurotransmitters, such as NE, serotonin or ATP (Gibbs et al., 2006; Gibbs, 2016). It is speculated that glycolytic metabolism can be also regulated by coordinated GPCR-coupled cAMP and Ca^2+^ signals or non-GPCR-coupled Ca^2+^ and cAMP signals (Bak et al., 2018). Pathologically, disturbance in glycogen homeostasis may decrease glycogenesis and increase glycogenolysis in AD. The glycogen synthesis may be diminished by overactivation of synthase kinase 3 (GSK3) which also promotes abnormal hyperphosphorylation of tau and neuroinflammation process (Beurel et al., 2015; Di et al., 2016; Rodríguez-Matellán et al., 2020). Besides, GSK3 activity may be diminished by cAMP-dependent PKA (Llorens-Marítin et al., 2014). On the other hand, exposure to β-amyloid augments the energy consumption of neurons. In such conditions, increased excitability seems to generate a temporary compensation mechanism in response to synaptic loss. Bass and colleagues argue that neuronal metabolic changes drive the depletion of glycogen reserves in the brain *via* elevated levels of Aβ and astrocytic A_2A_ receptors, ultimately resulting in neuronal hypoactivity as the disease progresses (Bass et al., 2015). Considering that the overactive A_2A_ receptor is sufficient to cause hippocampal-dependent cognitive impairments *via* PKA/cAMP/CREB signaling (Li et al., 2015) as well as able to affect transcription of genes related to neuroinflammation, angiogenesis and astrocytic reactivity (Paiva et al., 2019), the development of novel therapeutic target against astrocytic A_2A_ receptor-dependent mechanism seems to be rational in the treatment of the AD. *In vitro* study has demonstrated that A_2A_ receptor antagonism prevented Aβ-induced synaptic degeneration in hippocampus (Gomes et al., 2011). Additionally, the latest results from live-cell imaging of primary cortical astrocytes revealed that application of Aβ_25−35_ peptide can induce PMCA-mediated Ca^2+^ extrusion *via* cAMP signaling. Thus, ATP-dependent Ca^2+^ extrusion system seems to constitute a protective mechanism aimed to counterbalance the early effects of Ca^2+^ overload in the presence of neurotoxic Aβ oligomers. On the other hand, their increasing chronic aggregation may impair PMCA activity in astrocytes (Pham et al., 2021).

Additionally, astrocytic Ca^2+^ signal may generate a rapid astroglial response to the nearby autoreactive immune cells involved in immune-mediated demyelinating disease, called multiple sclerosis (MS). It is believed that this mechanism of intercellular communication may be linked to astrocytic purinergic activation and participation of ATP release to autoinflammation in the CNS (Bijelić et al., 2020). On the other hand, β_2_-adrenergic-dependent cAMP signaling in reactive astrocytes appears to be downregulated putatively as a result of complete loss of β_2_-adrenergic receptor immunoreactivity in the MS, what may contribute to disease pathology (De Keyser et al., 1999, 2004). Recently, amyotrophic lateral sclerosis (ALS)- and frontotemporal dementia (FTD)-linked TAR DNA-binding protein 43 (TDP-43) inclusions have been shown to affect cAMP and Ca^2+^ signaling in astrocytes, most likely due to the downregulation of β_2_-adrenergic receptors leading to dysregulated astroglial metabolism and disease progression (Velebit et al., 2020).

Similar to AD pathology, reduced glutamate transporters expression has been detected in neurotoxin-induced PD animal models (Holmer et al., 2005; Chung et al., 2008). It is worth noting that the ventral midbrain-derived astrocytes are highly susceptible to D2 receptor-induced Ca^2+^ signaling. They seem to be relatively insensitive to NE and exhibit significantly different Ca^2+^-related gene expression profile compared to telencephalic astrocytes (Ibáñez et al., 2019; Xin et al., 2019). In mice SNpc, AAV-mediated GLT1 knockdown elicited reactive astrogliosis, dysfunctional Ca^2+^ homeostasis (by altered expression of Ca^2+^ channels and Ca^2+^/calmodulin-dependent protein kinases) and DA neuronal death generating parkinsonian phenotypes (Zhang et al., 2020). Recent evidence has shown that GLT1 expression is regulated by cAMP and CREB and its internalization depends on Ca^2+^ mobilization driven mainly by the Na^+^/Ca^2+^ exchanger (Liu et al., 2016; Ibáñez et al., 2019). Together, oscillations in Ca^2+^ and cAMP may be involved at multiple levels in the regulation of glutamate uptake by astrocytes. Hence, accumulation of pathological changes may determine severity of the excitotoxicity in individual brain areas.

Prior post-mortem studies indicated abnormally lowered brain-derived neurotrophic factor (BDNF) level in brain tissue of patients with PD (Mogi et al., 1999). Several other evidence showed that BDNF genetic polymorphisms increase the risk of PD-related cognitive impairments (Karamohamed et al., 2005; Altmann et al., 2016). In animal PD models, neurotrophic factors supported the function of dopaminergic system, including prevention from degeneration of dopaminergic neurons and improvement of dopaminergic neurotransmission (Migliore et al., 2014; Palasz et al., 2020). Moreover, the comparable level of BDNF expression between astrocytes and neurons of human brain cortex suggests an equally significant role of astrocytic BDNF in maintaining their neuroprotective and neuroregenerative potential (Koppel et al., 2018). On the other hand, increasing evidence shows that dopaminergic protection depends on changes in astrocytic Ca^2+^ and cAMP levels (Jennings and Rusakov, 2016). For example, Koppel et al. reported that dopamine-induced BDNF upregulation is dependent on cAMP/CREB stimulation through β-adrenergic receptors in primary astroglia. Likewise, the activation of PI3K/Akt/CREB pathway *via* FLZ treatment increased GDNF production by astroglia and improved the function of dopaminergic neurons in mouse models of PD (Bao et al., 2020).

### Effect on Water Homeostasis

One of the main proteins responsible for maintaining cerebral water balance are aquaporins (AQPs), especially type 4 (AQP4). It is highly concentrated in glial endfoot membranes and forms a molecular complex with a transient receptor potential cation channel subfamily V member 4 (TRPV4; Benfenati et al., 2011; Lunde et al., 2015). Loss of these channels or their defective membrane assembly, for example during aging, leads to significant impairments in astrocyte polarity, which may exacerbate neurodegenerative progress (Valenza et al., 2020). For example, deletion of AQP4 exacerbated Aβ accumulation and atrophy of astrocytes in APP/PS mouse model of AD (Xu et al., 2015). AQP4 KO mice demonstrated increased 1-methyl-4-phenyl-1,2,3,6-tetrahydropyridine (MPTP) neurotoxicity toward dopamine neurons in the striatum. This included alterations in astroglial function and reduced GDNF synthesis (Fan et al., 2008). Another study reported the MPTP treatment led to upregulation of AQP4 expression in mouse substantia nigra. The MRI brain scanning revealed increased water accumulation in the substantia nigra of PD patients (Ofori et al., 2015) suggesting an implication of AQP4 in water imbalance in the PD brain. It has also been reported that the modulation of AQP4 may control inflammatory process and AQP4 dysfunction increased the production of IL1β and TNFα triggering microglial reactivity in the midbrain (Sun et al., 2016).

Song and Gunnarson pointed to the relationship between cAMP signaling in astrocyte water permeability and the extracellular potassium concentration. Uptake of K^+^ activated AQP4 *via* PKA-dependent pathway and led to water permeability in astrocytes. Prolonged increase in intracellular K^+^ due to K_ir_ channel prevented water permeability *via* Ca^2+^/calmodulin-dependent regulation (Song and Gunnarson, 2012). The relationship between PKA and calmodulin in the regulation of subcellular localization of AQP4 has also been documented (Kitchen et al., 2020). This supports the idea that disrupted Ca^2+^/cAMP signaling may be involved in altered astrocyte water permeability. The functional coupling between these signaling pathways seems to be involved in neuronal hyperactivity associated with cognitive deficits observed in AD models.

### Effect on Apolipoprotein E

Second messengers, such as Ca^2+^ and cAMP, orchestrate the release of signaling molecules, including a peptide, called apolipoprotein (ApoE; Kockx et al., 2007), which is primarily synthesized by astrocytes. This peptide is crucial for synthesis, degradation and removal of Aβ from the brain. *In vitro*, Aβ-upregulated cAMP levels *via* the classical pathway, G_s_-AC, leading to increased ApoE secretion and altered lipid trafficking in astrocytes (Igbavboa et al., 2006; Rossello et al., 2012). Changes in the lipid composition as a result of ApoE4 dysregulation led to pathological Ca^2+^ influx and Ca^2+^ hyperactivity in astrocytes (Larramona-Arcas et al., 2020). Compelling evidence suggests that polymorphic variants of APOE gene may correlate with the development of sporadic AD. The presence of the APOE4 allele greatly exacerbates the risk of the disease, while the presence of the APOE2 allele decreases the susceptibility to the disease (Yu et al., 2014; Fernandez et al., 2019). Similarly, current data also indicate various influences of APOE genotypes on αSyn aggregation and APOE4 may exacerbate a series of abnormalities characteristic of PD, such as behavioral disturbances, loss of neural connections and astrogliosis (Zhao et al., 2020).

Taken together, isoform-specific effect of ApoE and alterations in second messenger system may have profound consequences on cholesterol transport and neurotoxicity-induced synaptopathy (Veinbergs et al., 2002; Igbavboa et al., 2006; de Chaves and Narayanaswami, 2008).

## Concluding Remarks

**Figure 2:**
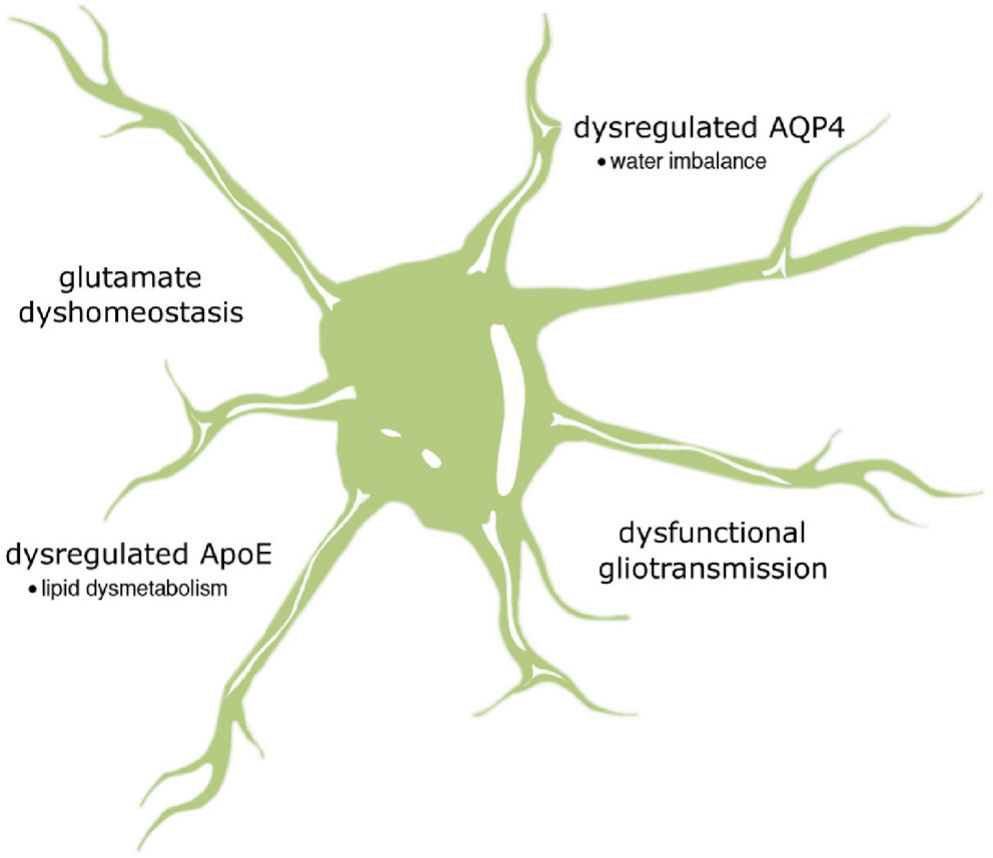
The summary of possible impact of astrocytic cAMP and Ca^2+^ signaling on neurodegenerative pathology. It is supposed that altered relationship between calcium and cAMP may be substantially relevant to the loss of gliotransmission and glutamate uptake as well as changes in the activity of two important proteins, AQP4 and ApoE, regulating water transport and lipid homeostasis, respectively.

It is commonly known that astrocytes can interact with multiple synapses to receive a vast amount of information. This information is next processed by a system of second messengers acting at different levels. Nonetheless, understanding their intricate interactions remains a challenge for the research environment. This review highlights the importance between cAMP and Ca^2+^ signaling in astrocytes, which modulates the surrounding microenvironment and synaptic plasticity. However, how these mutual relationships contribute to the mechanisms driving neurodegeneration is not fully understood. A growing body of evidence indicates that cAMP and Ca^2+^ interdependence can effect glutamate clearance from the synaptic cleft, gliotransmitter release and ion and water homeostasis. Even slight abnormalities in these mechanisms may have severe neuropathological consequences (Figure 2). The detailed mechanisms by which Ca^2+^/cAMP crosstalk in astrocytes affects pathological events leading to neurodegeneration are only beginning to emerge. In conclusion, further studies are essential for a precise understanding of Ca^2+^ and cAMP dynamics in astrocytes, taking into account the astrocyte region-specific phenotype and the regional susceptibility to synaptic damage and neuronal loss.

## Author Contributions

MS and TB conceived, designed the topic, and wrote the manuscript. Both authors contributed to manuscript revision, read, and approved the submitted version.

## Funding

This work was supported by the National Science Center (Narodowe Centrum Nauki) grant no. 2019/33/B/NZ4/00587 and by the Medical University of Lodz grant no. 503/6-086/-2/503-61-001.

## Conflict of Interest

The authors declare that the research was conducted in the absence of any commercial or financial relationships that could be construed as a potential conflict of interest.

## Publisher's Note

All claims expressed in this article are solely those of the authors and do not necessarily represent those of their affiliated organizations, or those of the publisher, the editors and the reviewers. Any product that may be evaluated in this article, or claim that may be made by its manufacturer, is not guaranteed or endorsed by the publisher.
